# Emerging Arthropod-Borne Infections in Temperate Regions: Comparative Synthesis Across Mosquitoes, Ticks, Sandflies, and Biting Midges

**DOI:** 10.3390/insects17030311

**Published:** 2026-03-13

**Authors:** Abdelaziz Touati, Takfarinas Idres, Christophe De Champs, Nosiba S. Basher

**Affiliations:** 1 Laboratoire d’Ecologie Microbienne, FSNV, Abderrahmane Mira University, Béjaia 06000, Algeria; abdelaziz.touati@univ-bejaia.dz; 2 Laboratory for Livestock Animal Production and Health Research, Rabie Bouchama National Veterinary School of Algiers, Algiers 16000, Algeria; t.idres@ensv.dz; 3 Laboratoire de Bactériologie-Virologie-Hygiène Hospitalière-Parasitologie-Mycologie, CHU Reims, Hôpital Robert Debré, Avenue du Général Koenig, CEDEX, 51092 Reims, France; cdechamps@chu-reims.fr; 4 Department of Biology, College of Science, Imam Mohammad Ibn Saud Islamic University (IMSIU), Riyadh 13318, Saudi Arabia

**Keywords:** One Health, climate variability/extremes, *Culicidae*, *Ixodidae*, *Psychodidae* (*Phlebotominae*), *Ceratopogonidae* (*Culicoides* spp.)

## Abstract

Arthropod-borne diseases are increasingly reported in temperate regions, reflecting the combined effects of climate variability, environmental change, and global movement of humans and animals. This review addresses how different arthropod vectors contribute to disease emergence in these regions and aims to identify shared and vector-specific drivers of transmission. A comparative synthesis of recent literature was conducted focusing on mosquitoes, ticks, sandflies, and biting midges, using a stage-based framework encompassing introduction, establishment and overwintering, seasonal amplification, and spillover to humans or animals. The analysis shows that although climate-related factors commonly facilitate expansion, the biological traits of each vector group strongly influence persistence, transmission dynamics, and detectability. Differences in overwintering capacity, host associations, and dispersal mechanisms result in distinct emergence pathways and surveillance challenges. The findings indicate that early warning indicators and control options are highly context dependent and cannot be applied uniformly across vector groups. This narrative review concludes that effective preparedness in temperate regions requires surveillance and intervention strategies tailored to specific vectors and ecological settings.

## Simple Summary

A brief synopsis. In temperate regions of Europe North America and beyond arthropod-borne diseases—transmitted by mosquitoes ticks sandflies and biting midges—are becoming more common. The conditions under which these vectors establish survive winters intensify seasonally and eventually spill over to cause disease in humans and animals are changing due to climate variability land-use change and the global movement of people and animals. Each vector group however reacts to these common pressures through unique biological mechanisms: biting midges benefit from the thermal buffering of livestock housing sandflies rely on fine-scale microclimate refugia ticks spread slowly but persistently into new areas and mosquitoes can quickly amplify transmission during warm seasons. This review compares emergence dynamics across all four vector groups by synthesizing literature from 2015 to 2025 using a four-stage framework: introduction establishment amplification and spillover. The analysis reveals that rather than being applied consistently early-warning indicators surveillance tactics and control options must be customized to each vectors unique constraints. Planning for readiness and the development of integrated One Health surveillance systems that can identify and address new vector-borne threats in temperate environments are directly impacted by these findings.

## 1. Introduction

Across the 2015–2025 temperate-emergence literature, warming trends, increased climate variability, and more frequent compound extremes (e.g., heatwaves with drought or anomalous precipitation) are framed as reshaping vector seasonality, pathogen replication, and winter survival, plausibly shortening extrinsic incubation and extending host–vector contact windows; causal attribution remains pathogen-, vector-, and surveillance-context dependent [1,2].

Land-use change and landscape restructuring further interact with climate to raise contact rates and restructure host communities. Urbanization tends to amplify container- and infrastructure-coupled mosquitoes (e.g., *Aedes* spp.) while fragmenting habitats and altering edge densities relevant to ticks; reforestation and wildlife recovery can also increase availability of competent hosts for tick populations and their pathogens [3,4].

Finally, temperate VBD emergence is increasingly driven by mobility and trade, which raise the rate of introductions of vectors and pathogens, seed repeated importations of pathogens into newly suitable areas, and move livestock reservoirs and products that couple to veterinary vector systems. For invasive mosquitoes in Europe, globalization-mediated introductions and rapid establishment of the *Aedes* invasive mosquito species have become a standing preparedness problem rather than a sporadic event, with recurrent local transmission episodes following pathogen importation [5,6].

Temperate regions are operationalized here using Köppen–Geiger climate classes dominated by “C” (temperate) and “D” (continental/cold) climates, including Mediterranean temperate subtypes (e.g., Csa/Csb) where many “temperate emergence” events occur at northern/altitudinal margins and in peri-urban microclimates. This operationalization is grounded in the high-resolution, widely used Köppen–Geiger update that enables consistent mapping of studies to climate strata and supports sensitivity analyses by excluding borderline arid or subtropical classes when needed [7].

We use standard definitions of vector competence and vectorial capacity (operationalized in Table 1 and in the stage model’s amplification logic) [8,9]. We use standard surveillance terms (active vs. passive; sentinel vs. syndromic) and define genomic surveillance as systematic pathogen sequencing from vectors, hosts, or clinical samples; operational denominators and common confounders are specified in Table 1 [10,11].

The review focuses on temperate-region emergence of four vector groups: mosquitoes (*Culicidae*), ticks (*Ixodidae*), sandflies (*Psychodidae: Phlebotominae*), and biting midges (*Culicoides* spp.) because each group represents a distinct constraint profile for overwintering, dispersal, host specificity, and control feasibility. Exemplars and mechanistic inference are drawn preferentially from temperate Europe and North America, where surveillance intensity is relatively high, but the scope includes temperate Asia and the Southern Hemisphere, where eligible data exist. Vector groups outside the scope (e.g., triatomines) are excluded unless directly relevant to a comparative methodological point [12,13]. Although several focal taxa are widespread within temperate zones (e.g., *Ixodes ricinus*, *Culex pipiens* complex, *Culicoides obsoletus* ensemble), the decision-relevant question is not continental-scale presence/absence, but rather how climate–land-use–mobility and host-community structure shift systems across emergence stages (introduction → establishment/overwintering → amplification → spillover) and modulate *local* hazard metrics (e.g., density of infected nymphs, vector infection rates, and season length) [14,15,16].

This narrative review uses a stage model (introduction → establishment/overwintering → amplification → spillover/burden) to compare mosquitoes, ticks, sandflies, and biting midges, asking how shared macro-drivers and vector-specific constraints gate stage transitions, which stage-aligned early-warning indicators are operationally actionable (and where surveillance fails), how genomics separates repeated introductions from local persistence, and which control responses are feasible in temperate contexts [6,17,18].

## 2. Methods (Transparent Narrative Review)

### 2.1. Search Approach and Sources (Databases + Institutional Sources; Search Blocks)

This article is a narrative review informed by structured searching of bibliographic databases to support a transparent, stage-based comparative synthesis across vector groups. We did not conduct a PRISMA-traceable scoping or systematic review workflow (e.g., formal duplicate screening, complete capture claims, or a PRISMA flow diagram), and the manuscript should be interpreted accordingly as an interpretive synthesis that prioritizes cross-taxon explanation over comprehensive enumeration of the literature [18,19].

The primary literature search covered 1 January 2015 through 31 December 2025 in PubMed/Medline, Scopus, and Web of Science Core Collection. Search logic used three modular blocks combined with Boolean operators: (A) vector block (mosquito* OR *Aedes* OR *Culex* OR tick* OR *Ixodes* OR sand fl* OR phlebotom* OR *Phlebotomus* OR *Culicoides* OR biting midge*), (B) temperate/emergence block (temperate OR “Köppen” OR “continental” OR “Mediterranean” OR expansion OR emergence OR establishment OR overwinter* OR diapause OR autochthonous OR outbreak), and (C) driver/surveillance block (climate OR heatwave OR drought OR land use OR urban* OR trade OR travel OR movement OR sentinel OR surveillance OR genom* OR sequencing). These searches were used to structure coverage across vector groups and emergence stages, but they are not presented as evidence of exhaustive capture [20]. We restricted sources to peer-reviewed journal articles indexed in PubMed/Medline, Scopus, or Web of Science, and excluded institutional reports and websites.

### 2.2. Eligibility Criteria (Temperate Emergence Evidence; Study Types; Tropical Exclusion Logic)

Eligible records were peer-reviewed journal articles (original studies, systematic reviews, or modeling studies with clearly stated assumptions) providing evidence relevant to temperate-region emergence in at least one focal vector group. To avoid over-generalization, included studies had to specify the relevant vector taxon (species/complex where available), pathogen, and temperate geographic setting, and to provide stage-relevant evidence (introduction, establishment/overwintering, amplification, or spillover/burden). Studies set exclusively in Köppen ‘A’ tropical climates were excluded unless they explicitly tested mechanistic constraints relevant to temperate boundary conditions (e.g., temperature thresholds, diapause/overwintering biology, or validated temperate suitability models) [18].

### 2.3. Data Charting and Coding (Required Coding Framework)

A standardized charting framework was used to organize included evidence across: geography and temperate classification; vector taxon; pathogen/lineage where reported; emergence stage (Introduction, Establishment/Overwintering, Amplification, Spillover/Burden); proposed drivers (climate variability/extremes, land use, mobility/trade, host community shifts, governance/control capacity); and evidence type (entomological, veterinary, clinical, serology, genomics). Quantitative statements were retained only when explicitly supported by the cited source and accompanied by clear denominators and diagnostic methods, and no meta-analysis was attempted [20]. Study locations (country/region), assigned Köppen–Geiger climate class, and the climatic covariates and temporal windows used for inference are summarized in Supplementary Table S1.

### 2.4. Synthesis Approach and Confidence Rubric

Synthesis proceeded as a structured narrative comparative analysis aligned to the stage model, with conservative language used to separate detection from transmission and burden. To enhance interpretability, key comparative claims were assigned qualitative confidence levels (high/moderate/low) based on triangulation across independent study designs and explicit acknowledgement of surveillance confounding, rather than formal risk-of-bias scoring or effect pooling [18,19].

## 3. Conceptual Framing: Stages of Emergence in Temperate Regions

### 3.1. Introduction

Introduction denotes the arrival of a vector and/or pathogen into a temperate locality via mobility, trade, animal movements, or aerial dispersal, without yet demonstrating multi-season persistence. In practice, introduction evidence is dominated by detection signals: interception records, isolated adult captures, first records from routine traps, or imported human/animal infections that could seed local transmission if competent vectors are present. The evidentiary pitfall is that detection is highly surveillance-dependent; hence, the operational task at this stage is not to infer transmission but to quantify propagule pressure and exposure opportunity (e.g., frequency of detections per trap-night; number of introductions per season; proximity to ports of entry; timing relative to thermal thresholds for development or extrinsic incubation) [21,22]. Consequently, even where vectors are widespread, intensified monitoring is justified when pathogen detection, seasonal phenology, or hazard indices shift, because these are the features that move a locality across stages and change intervention timing [23,24]

### 3.2. Establishment/Overwintering (Persistence Across Winters)

This stage is the central “temperate gate” because winter mortality and seasonal bottlenecks impose vector-specific constraints: diapause eggs in invasive *Aedes* spp.; overwintering adult females in *C. pipiens* and related taxa; multi-year life cycles and cold tolerance in ixodid ticks; and protected larval habitats or indoor/animal-shelter microrefugia for sandflies and some biting midges. Accordingly, establishment should be asserted only when evidence exceeds single-season detection and includes either (i) immature stages indicating local reproduction, (ii) phenological patterns consistent with resident populations, or (iii) direct overwinter survival observations under field or semi-field conditions [22,25].

Overwintering biology is not only a “cold constraint”; it is increasingly a microclimate constraint shaped by urbanization and habitat coupling. Urban heat islands and sheltered built environments can delay or disrupt diapause induction, extend host-seeking seasons, and alter winter survival trade-offs (e.g., longer autumn activity but lower survival in winter-like conditions), implying that “temperate suitability” may be driven by fine-scale thermal environments rather than regional means. For invasive mosquitoes, diapause timing and synchronization can be decisive for establishment at range margins; for ticks, buffered microhabitats (leaf litter, soil interface) and host availability can decouple winter survival from ambient air temperatures; for sandflies, peri-domestic refugia (animal housing, stone walls) can similarly create establishment opportunities beyond what coarse climate envelopes predict [26,27].

Because establishment is often asserted prematurely, we treat it as an evidence-graded claim: “suspected establishment” (repeated detections and local reproduction evidence) versus “confirmed establishment/overwintering” (direct overwinter survival or multi-year continuity under consistent surveillance). Semi-field studies demonstrating adult overwinter survival in sheltered microclimates illustrate why establishment can occur via behaviors not captured in conventional species accounts (e.g., adult persistence where only egg diapause was assumed), but these findings must be mapped carefully to local context and winter severity before extrapolation. Establishment in sandfly systems similarly benefits from phylogeographic and population-structure evidence distinguishing transient introductions from self-sustaining populations, particularly where entomological surveillance is sparse and clinical Leishmaniasis diagnoses can lag [25,28].

### 3.3. Amplification (Seasonal Build-Up of Vector Populations and Infection Prevalence)

Amplification denotes the process by which established vector populations and pathogen transmission cycles intensify seasonally (or episodically), increasing infection prevalence in vectors and reservoir/amplifying hosts and elevating spillover risk. This stage is where mechanistic quantities such as vectorial capacity and its temperature-sensitive components (biting rate, vector survival, extrinsic incubation, and vector density) become most informative, and where macro-drivers (warm anomalies, drought/flood sequences) often manifest as short-term changes in transmission potential rather than as simple range shifts. Because amplification is the proximate driver of outbreaks, it is also the stage at which early warning is most actionable, provided surveillance captures both vector abundance (e.g., females per trap-night; nymphs per unit area) and infection metrics (e.g., minimum infection rate per 1000 vectors; proportion positive by PCR), with explicit denominators and sampling effort [21,29].

Amplification pathways diverge sharply across vector taxa. Mosquito-borne systems often exhibit rapid, weather-responsive amplification because multivoltinism permits explosive density increases and because extrinsic incubation periods shorten nonlinearly with temperature; tick-borne systems more often produce “slow-burn” amplification because multi-year life cycles, host community structure, and seasonal synchrony govern nymphal infection hazard over longer horizons. Sandfly amplification is frequently focal and microclimate-driven, with household-level clustering shaped by reservoir host ecology and peri-domestic habitat suitability. Biting midge amplification is strongly coupled to livestock density and management, with potential for abrupt increases during favorable seasons and the added complexity of windborne dispersal that can shift infection foci over short periods. These contrasts justify using different leading indicators (e.g., mosquito infection rates and degree-day accumulation versus tick nymphal density and host seroprevalence versus livestock sentinel seroconversion for orbiviruses) [30,31].

### 3.4. Spillover/Burden (Human and Veterinary Outcomes)

We treat spillover/burden as the highest evidence rung, requiring clinical/veterinary signals linked to entomological/ecological corroboration and interpreted against diagnostic/case-definition changes (Table 1) [32,33].

### 3.5. Mapping Stages to an Evidence Ladder and to Actionable Early-Warning Signals

**Figure 1:**
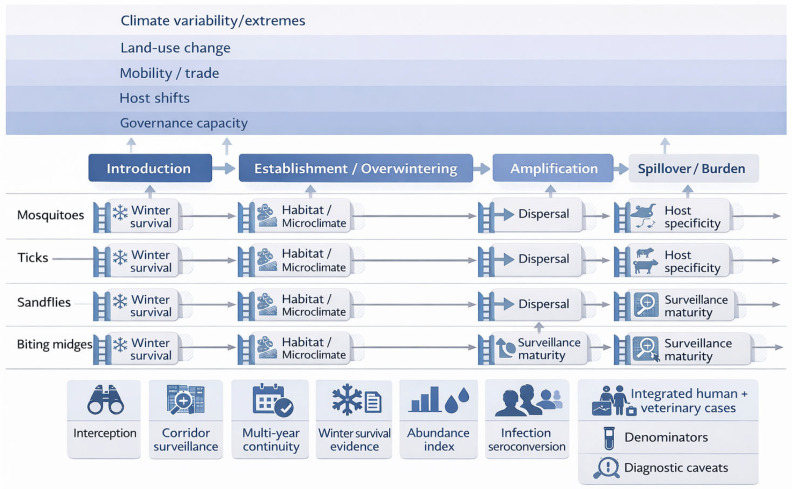
Stage model for temperate emergence in a multi-vector world, with driver mapping and intervention fit.

We grade claims using an evidence ladder (Table 1) that separates Detection, Competence, Transmission, and Burden, explicitly treating surveillance design and effort as determinants of apparent emergence (Figure 1) [34,35].

## 4. Mosquito-Borne Emergence Patterns and Drivers

Mosquito-borne emergence in temperate regions is best interpreted as two partially overlapping archetypes aligned to the stage framework: (i) *Culex*–bird enzootic cycles (notably West Nile virus and *Usutu* virus), in which amplification depends on seasonal vector abundance, avian community dynamics, and temperature-sensitive extrinsic incubation; and (ii) invasive *Aedes* systems (especially *Aedes albopictus*), in which establishment depends on diapause programming and winter microclimates, while spillover is typically triggered by repeated introduction of viraemic humans during periods when gonotrophic cycling and extrinsic incubation are sufficiently rapid. Importantly, *Culex* vectors are not “bird-only”: blood-meal syntheses and field studies show frequent mammalian feeding alongside avian feeding, supporting their bridge-vector role for zoonotic spillover when competent *Culex* populations co-occur with infected avian hosts [24,36,37].

In *Culex*–bird systems, temperate emergence often progresses from repeated detection (vector pools and avian hosts) to evidence consistent with local overwintering and onward seasonal amplification that precedes human/equine burden. Genomic and field evidence supporting overwintering has strengthened in the 2020–2025 period, including lineage documentation and seasonal persistence consistent with local maintenance in parts of southern Europe, while simultaneously underscoring that “presence in mosquitoes” is not synonymous with sustained transmission everywhere. Mechanistically, *C. pipiens* complex biology enables winter survival as diapausing adult females in sheltered hibernacula, decoupling viral persistence hypotheses from active winter biting and emphasizing the importance of springtime amplification conditions [36,38].

In invasive *Aedes* systems, the canonical temperate pathway is a two-step gate: (1) establishment with winter survival (classically via diapause eggs) and sufficient seasonal population growth; followed by (2) episodic introduction of virus through travel and mobility, producing focal transmission where temperature–biting–extrinsic incubation constraints are transiently relaxed. The temperate signal is therefore often a “lagged hazard” pattern: establishment occurs first, and measurable autochthonous dengue/chikungunya risk rises thereafter in a time-dependent manner, rather than tracking importations alone. This framing helps reconcile why multiple years of high importation can coincide with zero local transmission until establishment and microclimate thresholds align [39,40]. While diapause eggs remain the dominant overwintering form for *A. albopictus* in many temperate settings, evidence of adult persistence during winter in southern Europe has direct implications for surveillance timing (winter adult sampling becomes informative rather than futile) and for the length of the effective transmission season.

Population genetic signatures are consistent with multiple introductions and stepwise spread beyond major geographic barriers (e.g., beyond the Alps), implying that border interception alone is unlikely to prevent establishment once regional transport corridors seed new foci. Complementarily, high-sensitivity port and point-of-entry surveillance continues to detect propagules (e.g., eggs) in countries where establishment is not yet demonstrated, underscoring that “introduction pressure” can be high even when onward transmission remains absent [40,41].

Eco-climatic driver bundles differ systematically between the *Culex*–bird and invasive *Aedes* archetypes, and the strongest 2020–2025 contribution has been the operationalization of early warning using anomaly-based predictors. For WNV in Europe, explainable machine-learning models trained on multi-year data identify positive spring/summer temperature anomalies, reduced surface moisture (e.g., low NDWI), and antecedent dry winter conditions as major determinants of outbreak occurrence, with high discrimination when prospectively tested (AUC reported at 0.97 for 2018 and 0.93 for 2019). These approaches are decision-relevant because they convert climate variability into actionable lead time for intensified vector and host surveillance rather than post hoc attribution [16,42].

Land-use and host community context modulate these eco-climatic signals, complicating causal attribution and creating confounding with surveillance intensity (areas with better mosquito and bird surveillance can appear to “emerge” earlier). For WNV, analyses integrating climate, land use, and socio-economic change support the view that climate variability operates through environmentally mediated vector abundance and host contact structure (e.g., irrigated agriculture, wetlands, peri-urban mosaics), rather than as a standalone driver. This reinforces the stage-based interpretation: land use and host community structure are often amplification multipliers that determine whether establishment-level detection scales into measurable burden [43,44].

Surveillance maturity is comparatively high for mosquitoes in many temperate countries, but it is uneven across archetypes: WNV/USUV programs often have long-standing One Health components (mosquito trapping, avian surveillance, and, variably, veterinary and blood safety interfaces), whereas *Aedes*-borne arbovirus surveillance is frequently event-driven around imported cases and vector presence. Genomic surveillance is becoming an operational amplifier for inference about establishment and spread, as demonstrated by integrated One Health investigations that resolve WNV/USUV emergence dynamics [43,45]. Recent autochthonous dengue clusters in Europe (Table 2) indicate that response capacity—not just vector presence—can be rate-limiting for containing spillover [46,47,48].

Finally, human malaria and other mosquito-borne parasites provide a clarifying “confounding control” for temperate emergence: competent vectors can persist for decades without burden, and episodic transmission can be driven primarily by importation coupled with transiently permissive weather. In Florida (USA), autochthonous *Plasmodium vivax* infections in 2023 were supported by genomic evidence consistent with a likely single introduction and demonstrated the operational value of integrating clinical detection with genomics and entomology during response; broader qualitative synthesis of the 2023 U.S. events emphasizes containment and the improbability of sustained transmission under current conditions while acknowledging climatic permissiveness as a risk amplifier. Zoonotic filarial emergence in northern Europe (e.g., *Dirofilaria repens*) further underscores that veterinary and human signals can reveal establishment and endemicity in temperate settings even where human incidence is under-quantified, highlighting ascertainment bias as a persistent limitation [49,50,51].

**Table 2:** Exemplar mosquito-borne pathogens in temperate regions (2015–2025) and the highest evidence-ladder stage reached.

Temperate-Region Exemplar (Köppen Broadly C/D Where Applicable)	Pathogen (System)	Principal Mosquito Vector(s) Implicated	Highest Evidence-Ladder Stage Reached in Exemplar	Brief Evidence Basis (Stage Justification)
Europe (multiple temperate C/D regions)	WNV (enzootic *Culex*–bird)	*C. pipiens* complex (context-dependent local vectors)	**Burden**	Predictive models and multi-year analyses consistent with expanding outbreak suitability and realized outbreaks; key determinants include temperature anomalies and moisture indices, supporting actionable early warning [16,44].
Netherlands and NW Europe (temperate Cfb)	USUV (enzootic *Culex*–bird)	*C. pipiens* complex	**Transmission** (with human/avian impact variably documented by setting)	Field evidence supports overwintering in mosquitoes and integrated One Health reconstruction of arbovirus dynamics, strengthening inference beyond detection alone [43,52].
Mainland France (temperate to warm-temperate Cfb/Cfa)	DENV (invasive *Aedes*; focal autochthonous)	*A. albopictus*	**Transmission** (episodic clusters; some settings approach outbreak-scale burden)	Autochthonous dengue transmission reported with geographic extension and increased incident foci, consistent with establishment plus importation-driven spillover [48,53].
Italy (temperate Cfa/Cfb; central Italy exemplar 2024)	DENV (invasive *Aedes*)	*A. albopictus*	**Burden** (localized outbreak)	Large outbreak report (e.g., 86 cases in 2024 central Italy exemplar), consistent with sustained local transmission over weeks and substantial case ascertainment [47].
Italy (temperate Cfa/Cfb)	CHIKV (invasive *Aedes*)	*A. albopictus*	**Burden**	Documented outbreak-scale transmission consistent with established *A. albopictus* and conducive seasonal conditions (tempered by surveillance and diagnostic heterogeneity) [54,55].
Southeastern Australia (temperate riverine systems; 2022)	JEV (zoonotic amplification with pigs/birds)	*Culex annulirostris* and related vectors	**Burden**	Sentinel human case and widespread animal detections in 2022, with vector synthesis supporting plausible transmission ecology and control implications [56,57].
Florida, USA (humid subtropical Cfa; 2023)	*Plasmodium vivax* (human malaria re-introduction)	*Anopheles* spp. (local vectors; exemplar includes *Anopheles crucians*)	**Transmission** (contained)	Cluster of locally acquired cases with genomic evidence consistent with introduction(s) and response synthesis emphasizing containment and low likelihood of sustained transmission under current conditions [50,51].
Baltic countries (temperate Dfb/Cfb mix; emergence northward)	*Dirofilaria repens* (zoonotic filariasis)	Multiple mosquitoes (regional assemblages)	**Transmission** (established/endemic in dogs; human autochthonous cases documented)	Review evidence supports establishment/endemicity over a decade with One Health relevance; human incidence under-quantified, highlighting ascertainment bias [49].

## 5. Tick-Borne Emergence Patterns and Drivers

### 5.1. Range Shifts and Establishment: Climate Suitability Is Necessary but Not Sufficient

A consistent 2015–2025 signal across North America and Europe is the poleward and altitudinal expansion of key *Ixodes* vectors, including *Ixodes scapularis* and *I. ricinus*, with establishment defined operationally by repeated detection of multiple life stages across years, i.e., evidence compatible with overwintering and local reproduction rather than transient introduction on hosts [58]. In an emergence-zone analysis in Québec, Canada, passive surveillance data were leveraged to estimate a mean expansion speed of ~18 km·year^−1^ for I. scapularis, illustrating that establishment can be gradual yet directional at landscape scales [58]. More recent synthesis emphasizing 2020–2025 advances reinforces that climate suitability metrics (e.g., accumulated temperature/season length) plausibly enable establishment at higher latitudes, but host availability, land cover, and dispersal by deer and birds modulate realized spread and the spatial continuity of colonization [59].

Europe provides an analogous but heterogeneous pattern: warming trends and changing seasonal profiles are associated with changes in tick activity windows and geographic suitability for *I. ricinus*, yet local persistence is tightly constrained by near-ground humidity and vegetation structure that buffer desiccation and cold exposure [60]. This microclimate dependence creates patchy establishment mosaics (woodland edges, shaded understory, peri-urban refugia) that can be “invisible” to coarse climate grids and national case surveillance, complicating attribution of emergence to climate alone and increasing the value of near-surface environmental monitoring and standardized field sampling [61].

### 5.2. Amplification: Host Community Shifts, Wildlife Recovery, and Land Fragmentation

Following establishment, amplification (sustained enzootic cycling with measurable infection prevalence in questing ticks) depends on host community structure rather than tick presence alone. Large ungulates (e.g., deer) can be critical for adult female feeding success and thus tick population growth, but they are often poor reservoirs for *Borrelia burgdorferi* sensu lato; consequently, deer recovery can increase tick density without a proportional increase in nymphal infection prevalence. In contrast, small mammals—particularly rodents—are central because they both feed large numbers of larvae and act as key reservoirs for rodent-adapted *Borrelia* (e.g., *B. afzelii*), thereby shaping the density of infected nymphs through coupled effects on larval feeding success and pathogen amplification. Experimental and observational evidence in European *I. ricinus* systems shows that variation in rodent abundance can predict subsequent-year tick hazard metrics (DON/DIN), supporting explicit inclusion of rodent dynamics when interpreting “*Ixodes* abundance” drivers [15,62,63,64].

Land-use change in temperate regions, particularly forest fragmentation, suburban expansion, and the creation of edge habitats, reshapes host movement and community assembly in ways that can increase human–tick contact rates even when average tick density is not exceptional, thereby advancing the system from amplification to spillover [63]. Fine-scale studies in emergence settings underscore that residential–woodland ecotones and peri-domestic vegetation structure can sharply elevate tick density and infection prevalence over tens to hundreds of meters, creating “risk gradients” that are operationally relevant for prevention yet easily missed by coarse administrative reporting [65]. In Europe, comparable urban–rural contrasts may be weak or inconsistent when measured purely as density of nymphs (DON) or density of infected nymphs (DIN), because urban green space connectivity and local host use can sustain enzootic cycles; for example, DON values on the order of ~18–26 nymphs per 100 m^2^ and DIN on the order of ~1.8–2.7 per 100 m^2^ were observed across an urban–peri-urban–rural gradient in southern England, with limited evidence of monotonic urban attenuation [61].

### 5.3. Spillover and Burden: Lags, “Slow-Burn” Epidemiology, and Multi-Pathogen Complexity

Using integrated passive and active surveillance data in Ontario, Canada, a ~5-year delay was reported between I. scapularis population establishment signals and subsequent emergence of *B. burgdorferi* in ticks, an empirical expression of the establishment → amplification lag that can misclassify an area’s risk stage if surveillance focuses narrowly on short-term human case counts [66].

European tick-borne encephalitis (TBE) exemplifies how burden can expand through a combination of ecological suitability and surveillance/behavioral factors, with spatiotemporal spread documented across the EU/EEA and drivers frequently implicating seasonal temperature anomalies and land-use/host context while remaining sensitive to vaccination coverage and reporting heterogeneity [67]. National-scale analyses in the Nordic region likewise highlight that interannual variation in TBE burden can be associated with environmental covariates and human behavior, but causal attribution remains challenging because exposure, diagnosis, and prevention (notably vaccination) are not spatially uniform [68]. Systematic clinical syntheses emphasize that TBE burden in temperate Europe has operational implications for early warning because it can increase in established vector regions via shifting seasonality and microfocal transmission rather than through obvious vector invasions [69].

### 5.4. Ascertainment and Diagnostic Biases: Separating Emergence from Improved Detection

Ticks generate strong surveillance confounding because public health systems frequently rely on passive case notifications and passive tick submissions, both of which are sensitive to health-seeking behavior, clinician awareness, and changing case definitions; thus, apparent emergence can reflect intensified detection rather than a true increase in transmission [70]. In Canada, targeted reviews and methodological discussions underscore that passive surveillance underdetection of Lyme disease and that the geographic footprint of risk can outpace confirmed case recognition, particularly in newly colonized areas where laboratory testing practices and clinician suspicion lag behind ecological change [71]. A historical synthesis of Canadian tick and animal surveillance further highlights that inconsistent sampling designs and jurisdictional heterogeneity limit comparability through time, reinforcing the need to treat “trend” statements cautiously unless accompanied by stable denominators and standardized sampling effort [72].

Diagnostic uncertainty also complicates causal inference: serological cross-reactivity, changes in testing algorithms, and heterogeneous reporting of clinical syndromes can shift observed incidence independently of ecological drivers, and these issues are particularly salient for multi-pathogen tick systems in which co-circulation of bacterial, viral, and protozoal agents is common [69]. For ecological surveillance, reliance on single metrics (e.g., tick presence or nymph density alone) risks category errors; multi-component measures such as DON, nymph infection prevalence (NIP), and DIN better align with exposure potential but remain resource-intensive and can vary sharply by habitat and year [23]. Longitudinal datasets demonstrate that year-to-year variability in DIN can be driven more by fluctuations in nymph density than by changes in infection prevalence, reinforcing the importance of consistent field protocols and careful interpretation of short time series [73].

### 5.5. Beyond Ixodes: Introductions of Thermophilic Ticks and the Overwintering Question

While *Ixodes*-borne emergence dominates much of the temperate literature, 2015–2025 evidence also highlights episodic introductions and occasional establishment signals for more thermophilic ticks such as *Hyalomma* spp. in parts of Europe, raising the operational question of whether introductions remain transient or can transition to establishment/overwintering under increasingly permissive climate and microclimate conditions [74]. Reports of *Hyalomma rufipes* overwintering in temperate Europe provide a cautionary illustration: even if population persistence remains uncertain or localized, overwintering evidence compresses the stage model by shifting the system from repeated “introduction” events toward plausible establishment risk, with direct implications for early-warning strategies for Crimean–Congo hemorrhagic fever virus (CCHFV) and other agents associated with *Hyalomma* [75]. Veterinary-facing surveillance and targeted field sampling around likely introduction routes (migratory birds, transported livestock, equine movements) are therefore strategically important for detecting stage transitions early, particularly where human case surveillance would be insensitive until after amplification has occurred [76].

## 6. Sandfly-Borne Emergence Patterns and Drivers: Microclimate Refugia, Peri-Domestic Reservoirs, Fragmented Surveillance

Establishment at the temperate margin is increasingly evidenced by multi-year re-detection at the same sites, consistent with local persistence rather than repeated introductions. In Southwest Germany, *Phlebotomus mascittii* was captured during 2015–2018 across a large survey (176 sites) with 149 individuals, including detections at previously known sites and 15 newly identified sites; the authors explicitly interpret repeated presence at the same trapping locations over multiple years as indicating population stability (i.e., establishment signals even where competence remains uncertain). Notably, *Phlebotomus perniciosus*, a confirmed vector of *L. infantum* in Mediterranean settings, was only trapped once since 2001, underscoring that temperate emergence may be led by vectors with less-resolved competence profiles and therefore a weaker “detection → transmission” inference chain. Comparable “edge-of-range” persistence signals have been documented in neighboring temperate contexts (e.g., Austria) where phlebotomine occurrence is reported outside classical endemic cores, reinforcing the need to treat repeated, site-specific detection as an establishment hypothesis requiring targeted overwintering and competence confirmation rather than as proof of sustained transmission [77].

### 6.1. Microclimate Refugia as the Dominant “Gatekeeper” Mechanism in Temperate Settings

In sandfly systems, microclimate can dominate macroclimate because larval habitats (often organic-rich soil, cracks, and sheltered substrates) and adult resting sites experience buffered temperatures and humidity relative to weather stations or gridded climate products. Mechanistically, temperate persistence is most plausible where microrefugia prevent lethal winter exposure while permitting spring diapause termination and population rebuilding [78]. A recent physiologically anchored analysis explicitly links north-eastern distribution borders to the thermal environment of underground shelters, proposing that rabbit and rodent burrows at approximately −20 cm depth can enable overwintering at range margins, while deeper shelters (>−50 cm) may remain stably cold (mean < 10 °C) in ways that inhibit post-winter diapause activation in northern regions, an important caution that “more buffering” is not linearly beneficial if it suppresses seasonal reactivation cues [79]. Urban and peri-urban microhabitats (built structures, heat-retaining surfaces, basements, animal shelters) have similarly been treated as potential refugia that can decouple local suitability from broader climatic envelopes, especially near northern limits where modest changes in winter minima or shelter availability may nonlinearly shift persistence probability [80].

### 6.2. Climate Variability and Hydroclimate as Amplifiers Once Refugia Exist

Once establishment is possible, interannual climate variability, particularly drought and seasonal precipitation structure, can act as an amplification lever by altering larval substrate moisture, organic matter decomposition, and adult survival. In Northern Italy’s Emilia-Romagna lowlands, an entomological surveillance network originally designed for mosquito-borne arboviruses (West Nile/Usutu) captured 3022 sandflies during 2018–2021 (with >50% collected in 2021), and the authors associate the highest-abundance season with a comparatively drier spring–summer period, interpreting dry conditions as enhancing sandfly abundance and potentially the suitability of larval habitats. This study is operationally salient for temperate emergence because it demonstrates that amplification and pathogen detection can occur even in landscapes considered “less suitable” at a coarse scale, when favorable seasonal conditions align with microhabitat availability. In the Emilia-Romagna lowlands, molecular screening of >1500 female sandflies in 54 pools detected TOSV in 5 pools and *L. infantum* in 7 pools [81].

Forward-looking risk analyses for the Iberian Peninsula similarly project that changing climate suitability can expand transmission risk landscapes for dogs, reinforcing the expectation that temperate-fringe amplification will be episodic and climate-sensitive rather than smoothly trending and that attribution claims must remain conditional on model structure and input assumptions (e.g., how vectors and microclimate are parameterized) [82].

### 6.3. Peri-Domestic Reservoirs and Host-Community Coupling: Introduction Versus Local Maintenance

Temperate emergence is often initiated through pathogen introduction via movement of infected reservoir hosts, especially dogs for *L. infantum*, but the operational problem shifts once vectors become established: peri-domestic coupling can convert imported infection pressure into local transmission opportunities even when vector densities are patchy. In Switzerland a temperate setting where suitability is highly heterogeneous serologic screening of domestic animals illustrates both the interpretive challenges and surveillance value of companion-animal sampling: among 101 dogs and 126 cats, seropositivity was 3.0% (3/101) in dogs and 1.6% (2/126) in cats in one recent study, with the authors emphasizing Switzerland’s “low-density” sandfly context and the consequent difficulty of inferring autochthonous transmission without integrated entomological and travel-history data [83]. In newly endemic temperate-adjacent foci in Northern Italy, a One Health geostatistical framework linked entomological presence probability of *Ph. perniciosus* to autochthonous canine and human outcomes 142/839 (17%) sampling sets positive for *Ph. perniciosus* and an odds ratio of 2.66 (95% CI: 2.16–3.37) for autochthonous canine leishmaniosis per 10% increase in modeled vector-presence probability illustrating how host reservoirs and human burden can become spatially coupled once establishment is achieved [84].

### 6.4. Fragmented Surveillance and “Evidence Ladder” Slippage in Temperate Sandfly Systems

Compared with mosquitoes and many tick systems, temperate sandfly surveillance remains comparatively fragmented, often project-based, and biased toward presumed hotspots (south-facing valleys, peri-domestic animal holdings), creating a structural confounder: increasing detection may reflect intensified searching rather than true range expansion. Experimental work on *Leishmania* development in sandflies continues to underscore that “detection” is not equivalent to “transmission,” because successful establishment of transmissible infections depends on parasite–vector compatibility and within-vector developmental constraints; accordingly, temperate surveillance systems that rely heavily on pooled PCR without pairing to competence-relevant entomology risk systematic over-inference [85,86,87].

### 6.5. Sandfly-Borne Viruses: Microclimate-Sensitive Extrinsic Incubation and Operational Blind Spots

**Figure 2:**
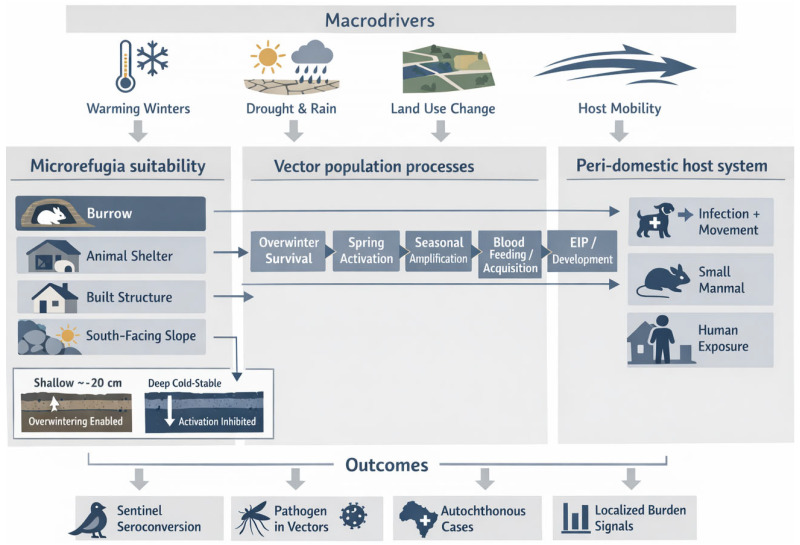
Microclimate refugia and peri-domestic coupling: a conceptual schematic for temperate sandfly emergence.

For phleboviruses such as TOSV, temperate emergence may be further shaped by temperature-sensitive extrinsic incubation and potential vertical/overwintering mechanisms, but the evidence base at temperate margins remains thinner than for *Leishmania* and is more susceptible to surveillance gaps because routine vector programs frequently prioritize mosquitoes (Figure 2). A recent mechanistic study estimated a TOSV extrinsic incubation period of 6 days in *Ph. perniciosus* and documented impacts on egg hatching time, reinforcing that small changes in thermal opportunity windows could alter onward transmission probability and that virus–vector interactions can feed back on vector life history in ways relevant to temperate phenology [88]. Temperate entomological surveys that detect phlebotomines but do not systematically test for phleboviruses therefore risk missing early amplification signals, especially where vectors are present but perceived pathogen risk is assumed low; this surveillance asymmetry has been explicitly flagged in temperate Germany, where repeated *P. mascittii* detection has outpaced competence resolution and pathogen-focused monitoring [89].

### 6.6. Implications for Operational Early Warning: From Microrefugia Mapping to Genomic Corroboration

Genomic epidemiology adds particular value in temperate settings where distinguishing importation from local circulation is central: in Northern Italy, whole-genome sequencing and epidemiologic analysis documented the emergence of an unusual *L. infantum*/*L. donovani* hybrid associated with atypical epidemiology, illustrating how genomics can identify emergence mechanisms (e.g., hybridization) that would be invisible to routine case counts and can materially alter risk assessment for temperate regions with substantial travel and animal movement [90].

## 7. Biting Midge–Borne Emergence Patterns and Drivers (Culicoides)

Among temperate-region vector-borne disease (VBD) systems, *Culicoides* biting midges (*Diptera*: *Ceratopogonidae*) are distinctive because their principal disease burdens are veterinary and trade-facing, yet their emergence dynamics are tightly coupled to climate variability, livestock geography, and animal-movement governance. In Europe and comparable temperate settings, the 2015–2025 evidence base most consistently links *Culicoides* to (i) recurrent *bluetongue virus* (BTV; *Orbivirus*, *Reoviridae*) incursions and re-emergence (e.g., BTV-8 in France from 2015), (ii) newly recognized epizootic hemorrhagic disease virus (EHDV; *Orbivirus*) emergence (EHDV-8 in Italy, 2022), and (iii) continued *Schmallenberg* virus (SBV; *Orthobunyavirus*, *Peribunyaviridae*) enzootic circulation with episodic recrudescence. In these systems, a recurring comparative theme is that “temperate emergence” is rarely a single-vector narrative: the same outbreak wave can be shaped by multiple *Culicoides* complexes (*C. imicola* in Mediterranean contexts; the *Obsoletus* and *Pulicaris* ensembles farther north), and by veterinary surveillance architectures that can detect transmission earlier than human health systems typically can [21,91,92,93,94].

Introduction (entry into temperate zones) in *Culicoides*-borne systems is frequently dominated by windborne dispersal interacting with animal movements and market connectivity, rather than by the ground-based diffusion typical of many tick-borne expansions. Trajectory- and risk-assessment studies during 2019–2025 operationalize the introduction step as the probability that infected midges are advected across ecological or political boundaries (including sea barriers), providing a mechanistically plausible explanation for punctuated, long-jump appearances of BTV serotypes in temperate locales. For example, windborne transport has been explicitly evaluated as a plausible introduction route for BTV-3 into Sicily from North Africa [95] and for *Culicoides* dispersal in emergent EHDV settings in France [96]; a quantitative risk assessment framework has also been applied to long-distance wind dispersal of *Culicoides* as an introduction pathway into mainland Europe (case study Sardinia) [97]. Importantly, animal-movement networks can then convert an introduction into a geographically distributed exposure landscape (amplification precursor), but movement restrictions alone cannot “block” a windborne vector, creating a governance asymmetry: trade and movement policy can reduce host-to-host connectivity while leaving airborne vector introduction risk largely intact [98,99].

Establishment and overwintering (temperate persistence) in *Culicoides* systems is operationally defined less by classical diapause physiology than by habitat coupling to livestock premises and winter microclimate buffering that sustains low-level adult activity and, consequently, a non-zero transmission potential. Indoor/outdoor trapping in temperate Europe has repeatedly shown that “vector-free” periods are shorter inside stables than outdoors, implying that establishment risk is shaped by animal housing and farm microclimates as much as by regional meteorology [100,101]. The most policy-salient recent evidence comes from intensive winter monitoring in livestock houses in Germany: across 960 trap catches, 32,377 *Culicoides* were collected and activity persisted at very low outdoor temperatures (autumn > 4 °C; winter > 0 °C), consistent with indoor thermal buffering (mean indoor temperature 1.2 K higher than outdoors; maximum observed indoor–outdoor difference 9.0 K) [102]. Flight-activity thresholds measured in UK field populations reinforce that small temperature differences near critical thresholds can determine whether vectors are airborne and biting (e.g., *Obsoletus* group activity detectable near ~4 °C in autumn and ~10 °C in spring; *Pulicaris* group near ~3 °C in autumn and ~12 °C in spring), underscoring why building microclimates can matter disproportionately for establishment [103]. Mechanistically, overwinter persistence remains a “multiple hypothesis” space in which vector activity in sheltered environments, prolonged host infectiousness, and vertical transmission pathways may all contribute [104].

Amplification (seasonal growth of transmission intensity) is consistently driven by the interaction of temperature-dependent vectorial capacity and high-density ruminant host landscapes, with land management and husbandry creating breeding opportunities (e.g., moist, organically enriched substrates) and concentrating blood-meal access. Long-term monitoring in the United Kingdom documented a ~40-day extension of the adult active period at one site, indicating that “season lengthening” is a measurable early warning signal for amplification potential, but also that responses are site-heterogeneous and an explicit warning against coarse, region-only climate metrics [105]. Mechanistic microclimate approaches in Denmark show how farm-level temperature fields can be translated into transmission parameters: estimated hourly microclimatic temperatures around all Danish cattle farms (2000–2016) were used to quantify spatial–temporal variability in the extrinsic incubation period (EIP) for SBV, and a related framework estimated infectious bites generated per BTV-infected host using microclimate at 22,004 farms combined with 1453 light-trap collections (2007–2016) [106]. Temperature-suitability modeling further supports a non-linear amplification window for BTV transmission potential in livestock (with suitability concentrated across intermediate temperatures and declining at extremes), providing a pathway to operational degree-day and threshold-based alerting rather than binary “vector present” reasoning [107]. At the landscape scale, surveillance-derived modeling of *Culicoides* abundance in mainland France illustrates how routine trapping data and temperature covariates can be integrated to forecast spatially heterogeneous amplification potential, enabling surveillance prioritization in high-risk livestock corridors [108]. Finally, once a novel serotype enters a temperate livestock network, amplification can be modulated by herd structure and density: post-epidemic seroepidemiology and risk-factor analyses for BTV-3 in Dutch dairy cattle explicitly quantify population exposure and identify farm-level correlates of infection risk (effect sizes as reported in the source), highlighting that “temperate amplification” is often a cattle-dominant phenomenon even when sheep contribute most to clinical signal visibility [109].

Spillover and burden in *Culicoides* systems are typically measured as veterinary clinical impact, reproductive losses, mortality, and trade disruption outcomes that can be substantial even when early phases are characterized only by subclinical circulation in cattle. The 2015 re-emergence of BTV-8 in France illustrates how cattle can sustain substantial subclinical circulation while sheep provide more conspicuous clinical signals (see Table 3 for prevalence estimates) [92]. More recently, BTV-3 emergence in western Germany was linked to detection of BTV-3 RNA in one pool of trapped *Culicoides* collected during ruminant infection reporting, while SBV RNA was found in many midge pools, an explicit demonstration of how entomological surveillance can move beyond “vector presence” toward transmission-relevant detection, while still stopping short of quantifying population-level burden without parallel animal outcome data [94]. In Belgium, BTV-3 was first detected in July 2024 and was associated with abortions and congenital central nervous system malformations in cattle, documenting clinically consequential transplacental outcomes in a temperate epidemic wave [110]; contemporaneous synthesis from Belgium emphasizes increased mortality and “knowledge gaps hindering an evidence-based control program,” highlighting a recurrent confounder in temperate emergence: apparent increases in burden can reflect both genuine transmission intensification and improved ascertainment during high-attention epidemic periods [111]. In parallel, EHDV-8 detection in Italy (Sardinia and Sicily, October–November 2022) provides a high-confidence example of introduction plus establishment risk at the temperate–Mediterranean interface, supported by genomic evidence of a North African origin (>99.9% nucleotide identity to Tunisia 2021 strains) [93].

Veterinary surveillance and control feasibility differ systematically from mosquito- and tick-borne temperate systems, because the dominant hosts are managed, the diagnostic chain is often centralized, and interventions (vaccination, movement governance, housing modifications) can be deployed rapidly while vector control in open landscapes remains difficult. A key surveillance advantage is that entomological monitoring for *Culicoides* is already embedded in many European control architectures to inform seasonality, targeted surveillance windows, and (in some jurisdictions) movement policy; continent-scale modeling of monthly adult presence probabilities for *C. imicola* and the *Obsoletus*/*Pulicaris* ensembles across nine European countries exemplifies how surveillance data can be translated into operational maps for targeted sampling and risk communication [112]. On control, vaccination remains the most scalable lever for reducing burden in livestock once establishment and amplification are plausible; for SBV, a decision-focused synthesis explicitly frames vaccination of naïve female animals before reproductive age as a potentially cost-effective risk-reduction strategy, particularly in settings with intermittent circulation and accumulating susceptible youngstock [113]. Where vaccination coverage is incomplete or delayed, housing-based interventions can reduce biting exposure: recent evaluation of vector-proof accommodation provides evidence that physical exclusion can meaningfully protect livestock from *Culicoides* bites under temperate farm conditions, supporting a feasible “local control” option even when landscape-scale vector suppression is unrealistic [114].

Table 3 summarizes the vector–host–pathogen triangle and where the earliest actionable surveillance leverage points sit for major temperate *Culicoides* systems.

**Table 3:** *Culicoides* vector–host–pathogen triangle and surveillance leverage points in temperate emergence.

Representative Temperate System (2015–2025 Exemplars)	Vector Node (Typical Temperate Vectors; Constraints)	Host Node (Dominant Hosts; Ecological Coupling)	Pathogen Node (Features Influencing Stage Progression)	Surveillance Leverage Points (Earliest Actionable Signals by Node; Examples)
BTV-8 re-emergence (temperate France, 2015 onward) [92]	*C. imicola* (Mediterranean) and *Obsoletus*/*Pulicaris* ensembles (temperate); winter persistence favored by indoor refugia and short/porous “vector-free” periods [100]	Cattle often have high infection/seroprevalence with limited clinical signal; sheep contribute conspicuous clinical detection; strong farm-building coupling	Multiple serotypes; transmission intensity is tightly temperature-dependent; potential vertical transmission complicates overwintering inference [104]	Host node: syndromic monitoring (sheep clinical signs; cattle bulk milk/serology); targeted youngstock serosurveys. Pathogen node: RT-qPCR confirmation + serotyping; sequencing for strain continuity. Vector node: routine UV light-trap indices (females/trap-night) + seasonality to inform surveillance windows and movement policy [112].
BTV-3 emergence (northwestern Europe, 2023–2025; e.g., Germany/Belgium/Netherlands/Portugal) [94,109,111,115]	Predominantly *Obsoletus*/*Pulicaris* ensembles in temperate areas; high sensitivity to microclimate near flight thresholds [103]	Dairy cattle density and farm connectivity can structure amplification (risk factors as reported); sheep frequently provide an early clinical signal	Novel serotype introductions can outpace population immunity; strong incentive for rapid genomic surveillance to track spread and reassortment	Pathogen node (early): pooled *Culicoides* RT-qPCR during rising farm cases (supports transmission-relevant detection) [94]. Host node: bulk milk screening, mortality/production syndromics, targeted serology to quantify exposure; reproductive-event monitoring. Vector node: phenology/season-length monitoring as an early amplification indicator [105]
EHDV-8 emergence (Italy, 2022; Mediterranean–temperate interface) [93]	Likely *C. imicola* plus temperate ensembles were competent; introduction plausibly shaped by windborne dispersal and coastal meteorology [96]	Cattle clinically affected; wildlife (e.g., cervids) may contribute to silent circulation and detection challenges (context-dependent)	Genomic origin tracing can strongly support introduction inference (>99.9% identity to Tunisia 2021 strains)	Pathogen node: rapid RT-qPCR confirmation + whole-genome sequencing to infer origin and monitor spread [93]. Host node: syndromic surveillance in cattle (fever, oral lesions) plus targeted wildlife health reporting where relevant. Vector node: targeted trapping along coastal entry corridors during high-risk wind periods.
SBV enzootic circulation with episodic recrudescence (temperate Europe, 2015–2025) [106,116]	Primarily *Obsoletus* complex; winter/early spring activity can occur in and around stables [101]	Ruminant reproduction outcomes (abortions, congenital malformations) provide high-signal sentinel events; herd immunity waxing/waning drives periodicity	Short EIP at favorable temperatures; microclimate models enable fine-scale estimation of transmission potential	Host node (earliest): reproductive surveillance (abortion/malformation clusters) and serology in youngstock to detect immunity gaps. Pathogen node: RT-qPCR in clinical specimens and (where used) pooled vector testing. Vector node: microclimate-driven EIP alerting using farm-level temperature fields as a pre-season risk signal [106]

## 8. Cross-Vector Comparison: Analytic Payoff Across Drivers, Constraints, Surveillance, and Control

### 8.1. Shared Drivers

Climate variability and extremes are a common accelerator, but act through vector-specific bottlenecks. For mosquitoes, anomalous warmth and hydroclimatic variability can compress extrinsic incubation and increase vector–host contact, yielding sharp seasonal amplification when competent vectors and susceptible hosts co-occur [117]. For ticks, warming more often translates into longer seasonal activity windows and faster development rates, which increase cumulative hazard without necessarily producing outbreak-like discontinuities in incidence [59].

### 8.2. Distinct Constraints (Why the Same Driver Yields Different Emergence Trajectories)

Across taxa, winter persistence is the key ‘temperate gate,’ but it is expressed through different mechanisms (*Aedes* egg diapause and urban microclimates; *Ixodes* humidity-buffered microhabitats and host availability; sandfly refugia; *Culicoides* activity buffered by livestock housing), so the same driver produces different stage trajectories [14,59,118].

Dispersal mode and host specificity further differentiate the “shape” of emergence signals. *A. albopictus* spreads primarily via human-mediated transport (and then local diffusion), producing stepwise geographic expansion and concentrated peri-domestic risk where governance can be fragmented (private properties, heterogeneous compliance) [119]. Ticks are dispersal-limited at fine scales but can be moved by hosts; consequently, range change is often inferred from creeping shifts in submitted ticks and modeled suitability rather than abrupt colonization fronts [120]. Sandflies can disperse locally, but establishment at temperate edges is strongly contingent on suitable resting/breeding microhabitats and reservoir availability, yielding mosaic risk surfaces that are difficult to cover with sparse surveillance [121]. *Culicoides* can disperse both locally and wind-assisted, enabling rapid multi-farm spread once virus circulation is established in livestock networks; thus, introduction can be hard to prevent, but amplification can be measurably tracked through veterinary diagnostics and entomological pool testing [122].

### 8.3. Control Feasibility (What Works Operationally in Temperate Contexts, and Where Expectations Should Be Constrained)

#### 8.3.1. Policy, Feasibility, and Control Recommendations in Temperate Regions: Stage-Based Priorities with Vector-Specific Levers

Control feasibility in temperate regions is highest when interventions are explicitly aligned to (i) the emergence stage and (ii) the vector’s limiting constraint. Prevention is most cost-effective during introduction/early establishment, but only if early-warning indicators are operationally credible and governance can sustain routine action during “quiet” periods. Evidence that integrated vector management (IVM) can be evaluated at a multi-year scale in temperate settings supports a shift from reactive outbreak control to audited, stage-based preparedness (high confidence that multi-year evaluation is feasible; moderate confidence for generalizing effect sizes across contexts) [123].

#### 8.3.2. Vector-Specific Feasibility and Priority Actions (Temperate Settings)

- Mosquitoes (*Aedes*; *Culex*–WNV systems): Integrated *Aedes* control is feasible but constrained by private-property access/compliance; program options include source reduction, targeted adult control, and evaluated integrated approaches, including SIT within integrated programs [124,125,126]. In *Culex*–WNV systems, multi-year surveillance–control can reduce *C. pipiens* counts (reported RR 0.66), but burden effects depend on timing relative to seasonal amplification and climate variability [123].
- Ticks: Landscape-scale suppression is constrained by diffuse habitats and wildlife hosts; deer management can reduce nymphal density in some contexts, but is complex and long-horizon [127]. Prioritize exposure mitigation, surveillance designs that track establishment/amplification (DON/NIP/DIN with stable denominators), and prevention tools where available (e.g., vaccination for TBE in parts of Europe) [128].
- Sandflies (northern borders): Frame control as peri-domestic risk management integrating reservoir-focused veterinary actions and microclimate-informed focal measures; treat establishment as plausible in newly detected temperate locations and prioritize early veterinary–entomological integration [77]. A practical lever in zoonotic *L. infantum* systems is insecticide-impregnated dog collars that reduce sandfly blood-feeding over long durations [129].
- *Culicoides*/livestock viruses: Because introduction can be windborne and outbreaks can escalate rapidly, vaccination and movement governance usually deliver the largest risk reduction; farm-level housing/biosecurity can reduce biting exposure when needed [130]. During the Dutch BTV-3 outbreak, surveillance outputs were used to anticipate overwintering and trigger vaccination/movement actions; vaccination can reduce mortality even when it does not fully prevent infection, so coverage/timing/effectiveness are core early-warning indicators for next-season risk (Table 4) [131,132].

## 9. Discussion

### 9.1. Integrated Interpretation Across Taxa: Where Shared-Driver Narratives Mislead

For example, the unusually early West Nile fever (WNF) transmission season in Europe in 2018 used as a cautionary benchmark for early seasonal onset, illustrated that small shifts in timing can outpace blood safety and vector-control mobilization even where endemic transmission already exists (high confidence for timing shift; moderate confidence for attribution to specific meteorological mechanisms without local counterfactuals) [133].

### 9.2. Implications for Integrated Surveillance (One Health): A Minimal Package and Interoperability Requirements

A minimal One Health “integrated package” for temperate multi-vector systems can be specified as five interoperable components: (i) hazard monitoring (temperature, precipitation, humidity and derived indices) at operationally relevant spatial resolution; (ii) standardized vector surveillance (sentinel trapping networks and event-driven expansion) with pathogen screening; (iii) animal surveillance calibrated to reservoir/amplifier roles (e.g., birds/horses for WNV; ruminants for bluetongue); (iv) human surveillance combining case-based notification with syndromic triggers in high-risk seasons; and (v) genomic surveillance with predefined decision uses (lineage replacement, introduction tracking, antimicrobial/insecticide resistance markers where relevant). The key operational point is that genomics should not be “added on”, but embedded with defined turnaround times and sampling frames aligned to decision points (moderate-to-high confidence depending on sequencing capacity and governance) [134].

Interoperability is the dominant constraint once the minimal package exists: without shared geospatial identifiers, harmonized metadata (trap type, trap-night denominators, diagnostic assays, case definitions), and governance rules for cross-sector data access, integrated surveillance becomes a collection of parallel systems that cannot support early warning. Multi-vector, multi-host modeling frameworks highlight that the parameters driving invasion and amplification differ by habitat and host structure, reinforcing the need to chart surveillance outputs into comparable fields (vector abundance indices, host seroprevalence, lineage frequencies) rather than siloed metrics that are not commensurable across sectors (moderate confidence for model-guided prioritization; lower confidence for transferability when local host community data are sparse) [135].

### 9.3. Research Agenda (2025–2035): Cross-Cutting, Vector-Specific, and Implementation Science Priorities

Priorities for 2025–2035 are to (i) move from climate association to decision-grade attribution that explicitly resolves microclimate and human behavior and declares uncertainty from surveillance heterogeneity/host immunity (e.g., WNV framed for blood-safety implications) [2]; (ii) use genomics as hypothesis-testing for stage transitions (introduction vs. overwintering; single vs. multiple introductions) [136]; and (iii) generate implementation evidence on which integrated surveillance–control packages are deliverable, affordable, and sustainable by emergence stage, building on multi-year evaluation of IVM in temperate settings [123].

## 10. Limitations

As a narrative review (Methods, Section 2), selection bias and incomplete capture are possible; the synthesis is intended as a mechanistic comparative framework rather than an exhaustive census.

Publication bias is a material constraint on inference in temperate emergence: the peer-reviewed literature disproportionately reflects outbreak investigations, high-profile invasions, and well-instrumented settings, whereas endemic “low-noise” transmission, programmatic failures, and null intervention results are less consistently published. This interacts with country-level differences in preparedness and reporting completeness, raising the likelihood that apparent temporal increases partly reflect improved detection, political incentives to report, or shifting research attention, rather than a commensurate change in underlying transmission intensity [137,138].

Geographic bias is also likely. Temperate emergence evidence is densest where routine surveillance outputs and interoperable datasets are publicly available, particularly within parts of Europe and North America, which can over-weight inferences about drivers, early-warning signals, and control feasibility toward those governance and health-system contexts. Even within Europe, the availability and granularity of surveillance outputs vary substantially across countries and pathogens, constraining comparability and potentially inflating cross-national contrasts in “emergence” that are in part artifacts of heterogeneous observability [139,140,141].

Definitional ambiguity introduces additional uncertainty, particularly at stage boundaries (introduction vs. establishment/overwintering; amplification vs. *spillover*/*burden*) and in what constitutes “temperate” in practice when microclimate refugia enable localized persistence within broadly temperate Köppen classes. Cross-country differences in clinical case definitions, testing indications, and reporting rules can yield non-equivalent denominators for incidence, hospitalization, and seroprevalence, limiting the interpretability of apparent spatial gradients and temporal trends even when surveillance is notionally harmonized [142,143].

Finally, burden estimation is often weakest exactly where early warning is most desired. Several temperate emergence pathways can proceed via silent or subclinical circulation in animal hosts (and sometimes in vectors) before overt clinical signals emerge, while vaccination policy, herd immunity, and trade-related animal movements can decouple observed outbreak size from underlying transmission pressure. Consequently, even robust entomological or veterinary detections may not translate into immediate, attributable human or livestock burden, and cross-vector comparisons of “control success” remain tentative when outcome metrics differ (e.g., reduced vector indices vs. reduced incidence vs. avoided economic losses) [144,145,146].

## 11. Conclusions

Five comparative takeaways are decision-relevant. First, temperature trends and extremes are not a uniform “risk multiplier”: they can lengthen vector seasons and alter within-season dynamics, but the same anomaly can yield divergent effects depending on vector life history, habitat coupling, and local microclimate. Second, rapid-amplification systems (many mosquito–arbovirus cycles) disproportionately generate pulse emergence signals that align with anomalous eco-climatic sequences and can be forecasted probabilistically. Third, ticks typically produce creeping emergence range expansion and rising encounter rates that may precede discernible human burden, so early warning depends less on outbreak forecasting and more on sustained longitudinal indicators. Fourth, sandfly-borne threats at temperate margins are often governed by fine-scale refugia and peri-domestic transmission opportunities, creating high sensitivity to surveillance blind spots and misclassification of “introduction” versus “establishment” in fragmented monitoring landscapes. Fifth, *Culicoides*-borne emergence remains uniquely “operationally legible” in temperate regions because veterinary systems can couple movement controls, vaccination policy, and vector monitoring to interrupt amplification.

## Figures and Tables

**Table 1:** Evidence ladder used to grade claims across emergence stages.

Evidence Rung	Operational Criterion (Minimum)	Typical Data Streams (with Denominators)	High-Frequency Confounders to Declare Explicitly
**Detection**	Verified presence of vector and/or pathogen nucleic acid/antigen in a sample from a temperate locality	Trap captures (vectors per trap-night), tick drags (nymphs per 100 m^2^), midge light traps (females per trap-night), first records with georeferenced effort; pathogen detection in vectors/hosts with n tested	Surveillance expansion, trap placement bias, taxonomic misidentification, lab contamination, uneven effort across years
**Competence**	Demonstrated ability of a local vector population/species to acquire, support replication, and transmit under experimental conditions	Vector competence assays with infection/dissemination/transmission rates and n; temperature regime (°C) and gonotrophic timing	Non-representative colonized strains, unrealistic temperatures, microbiome effects, assay endpoint inconsistency
**Transmission**	Evidence of local transmission cycle (enzootic or livestock) consistent with vector biology and seasonality	Infected field vectors with infection rate estimates; sentinel seroconversion (%, n); temporally concordant animal cases; genomic clustering consistent with local circulation	Imported infections, serologic cross-reactivity, shifting diagnostics/case definitions, spatial misattribution of exposure
**Burden**	Quantified population-level impact in humans and/or animals	Incidence per 100,000 with case definitions; hospitalization/CFR; livestock losses with denominators; repeated seasonal recurrence	Under-ascertainment, healthcare access changes, awareness effects, reporting artefacts, changes in testing volumes

**Table 4:** Master comparative matrix: vector group × emergence stage × best indicators × most actionable interventions (temperate contexts).

Vector Group	Emergence Stage	Best Early-Warning Indicators (Stage-Aligned; Examples)	Most Actionable Interventions (Temperate Feasibility)
Mosquitoes (*Aedes*, *Culex*)	Introduction	Imported case pressure (incidence per 100,000 among travelers; spatiotemporal clustering); detection of invasive vectors in transport corridors; genomic evidence of repeated introductions vs. persistence. insecticide-resistance markers where monitored	Point-of-entry risk communication; rapid peri-focal vector surveys; trigger-based preparedness plans (larval habitat mapping; response staffing)
	Establishment/Overwintering	Recurrent early-season detections (trap-night indices); evidence of overwintered cohorts (early-season adult/egg detections); microclimate suitability signals (urban heat island/refugia)	Source reduction and container management; targeted larviciding in persistent habitats; enforcement/access strategies for private properties. winter preparedness audits
	Amplification	Vector abundance anomalies (per trap-night); pathogen detection in mosquito pools; sentinel animal signals where used; climate anomaly triggers linked to vectorial capacity proxies	Pre-emptive larviciding/adulticiding in defined risk polygons; integrated vector management packages; community engagement and compliance operations
	Spillover/Burden	Autochthonous case confirmation; genomic clustering consistent with local transmission; spatiotemporal concordance of cases with entomological indices	Incident management for outbreak response; peri-focal adult control; clinical/diagnostic advisories; blood safety measures where relevant
Ticks (*Ixodes* spp.)	Introduction	Novel detections at leading edges (passive submissions calibrated to effort); modeled suitability expansion corroborated by field sampling	Standardized sentinel sampling transects; public reporting systems with denominator-aware analytics; clinician awareness in frontier zones
	Establishment/Overwintering	Persistent multi-year presence across life stages; nymph density trends under standardized protocols; degree-day/land-cover metrics consistent with lifecycle completion	Habitat management pilots; targeted host management where feasible; structured evaluation of interventions (pre/post hazard metrics)
	Amplification	Rising infection prevalence in ticks (with denominators); increasing human exposure proxies (bite consultations; seroconversion in defined cohorts) adjusted for reporting changes	Exposure mitigation campaigns; acaricide strategies in high-risk microhabitats; occupational risk programs; integrated wildlife–landscape interventions where politically feasible
	Spillover/Burden	Clinically confirmed case incidence trends (per 100,000) with stable definitions; severe outcome monitoring; spatial concordance with entomological hazard	Improve diagnostics and reporting; targeted prevention in high-risk groups; long-horizon risk reduction via land-use and wildlife management policies
Sandflies (*Phlebotomus* spp.)	Introduction	First detections in northern-edge surveys; refugia mapping (built/rocky/peri-domestic shelters); updated distribution syntheses to reduce misclassification	Establish standardized trapping and taxonomic capacity; microhabitat-focused surveillance at likely refugia; reservoir screening in high-risk import zones
	Establishment/Overwintering	Repeated detections across seasons; evidence of local breeding/resting sites; microclimate stability within refugia	Targeted residual insecticide in resting sites (where appropriate); housing/peridomestic modifications; prioritize reservoir-focused prevention
	Amplification	Rising canine seroprevalence/clinical detection for *Leishmania infantum*; occasional pathogen detection in vectors; spatial expansion of vector abundance	Reservoir interventions (insecticide-treated dog collars; dog management); intensified entomological and veterinary surveillance; targeted focal control around clusters
	Spillover/Burden	Autochthonous human/animal cases with exposure histories; molecular typing consistent with local acquisition	Integrated case–vector response; strengthen clinical pathways; sustained reservoir control in emerging foci
Biting midges (*Culicoides* spp.)	Introduction	Veterinary notifications with rapid typing; whole-genome sequencing of virus; early-season serology in sentinel herds were used	Movement controls and pre-movement testing where applicable; surge diagnostic capacity; deploy trapping/pool testing in at-risk corridors
	Establishment/Overwintering	Off-season or early-season detection signals suggesting persistence (e.g., midge pool positives; early seroconversions); evidence of vector activity in housing	Routine midge surveillance at sentinel farms; biosecurity in livestock housing; preparedness vaccination planning (where vaccines exist/are authorized)
	Amplification	Rapid increase in farm-level positives; midge pool infection signals; modeled spread consistent with vector seasonality; vaccination coverage gaps	Vaccination campaigns (timing/coverage optimization); targeted communication to producers; enhanced outbreak analytics using farm network data
	Spillover/Burden	Excess mortality/production losses; clinical syndromic trends in ruminants with laboratory confirmation; assessment of vaccine effectiveness under field conditions	Sustain/expand vaccination; refine strain-matched vaccines; post-season evaluation (effectiveness, coverage, residual transmission) to inform next-season readiness

## Data Availability

No new data were created or analyzed in this study. Data sharing is not applicable to this article.

## Supplementary Materials

The following supporting information can be downloaded at: https://www.mdpi.com/article/10.3390/insects17030311/s1, Table S1: Study locations (country/region), assigned Köppen–Geiger climate class (approximate; see Beck et al., 2018) [7], and the climatic covariates and temporal windows used for inference. References [147,148,149,150,151] has been cited in Supplementary Materials file.

## Abbreviations

AUC	Area Under the Curve
BTV	Bluetongue Virus
CCHFV	Crimean–Congo Hemorrhagic Fever Virus
CFR	Case Fatality Rate
CHIKV	Chikungunya Virus
DIN	Density of Infected Nymphs
DENV	Dengue Virus
DON	Density of Nymphs
ECDC	European Centre for Disease Prevention and Control
EHDV	Epizootic Hemorrhagic Disease Virus
EIP	Extrinsic Incubation Period
FAO	Food and Agriculture Organization of the United Nations
JEV	Japanese Encephalitis Virus
MIR	Minimum Infection Rate
NDWI	Normalized Difference Water Index
PCR	Polymerase Chain Reaction
PRISMA	Preferred Reporting Items for Systematic Reviews and Meta-Analyses
PRISMA-ScR	PRISMA Extension for Scoping Reviews
SBV	Schmallenberg Virus
TBE	Tick-Borne Encephalitis
TOSV	Toscana Virus
USUV	Usutu Virus
VBD	Vector-Borne Disease
WGS	Whole Genome Sequencing
WNV	West Nile Virus
WOAH	World Organization for Animal Health
