# Supplementary material for: Emerging Arthropod-Borne Infections in Temperate Regions: Comparative Synthesis Across Mosquitoes, Ticks, Sandflies, and Biting Midges
Source: Insects. 2026 Mar 13;17(3):311. doi: 10.3390/insects17030311 (PMC13026707; doi:10.3390/insects17030311)
Supplement: Supplementary file 1 [file insects-17-00311-s001.zip › insects-4088137-supplementary.pdf]

Supplementary Table S1

Study locations (country/region), assigned Köppen–Geiger climate class (approximate; see Beck et al., 2018) [1], and the climatic covariates and temporal windows used for inference.

Vector group	Vector	Pathogen/system	Study location (country/region)	Köppen–Geiger climate class (code)	Climatic covariates used (as reported)	Temporal window/lag	Relevance to this review (stage model/context)	Ref
Mosquito	Culex pipiens complex	West Nile virus	Europe (multi-country)	Mixed temperate (Cfb/Dfb/Csa/Cfa)	Eco-climatic predictors (temperature/moisture anomalies; environmental indices) in outbreak prediction	seasonal/annual (as reported)	Amplification early warning (climate → outbreak probability)	[2]
Mosquito	Culex pipiens	West Nile virus	Italy (Emilia-Romagna)	Cfa (humid subtropical, Po Valley) / Cfb (uplands)	Meteorological + environmental drivers of mosquito infection prevalence (time series)	multi-year	Amplification (infection prevalence responds to the meteo-environment)	[3]
Mosquito	Culex pipiens	West Nile virus lineage 2	Spain (NE)	Csa/Csb (mediterranean) + Cfb (north)	Seasonal persistence across years (climate/seasonality context)	winter; seasonal/annual (as reported)	Establishment/overwintering inference	[4]
Mosquito	Culex pipiens	West Nile virus (vector ecology)	Italy (Central)	Cfa (humid subtropical, Po Valley) / Cfb (uplands)	Characterization of overwintering sites (hibernacula microenvironment)	winter; experimental (temperature regimes)	Overwintering biology (mechanistic specificity)	[5]
Mosquito	Aedes albopictus	Establishment risk	Urban temperate Europe	Mixed temperate (Cfb/Dfb/Csa/Cfa)	Microclimatic winter conditions (urban thermal buffering)	winter; seasonal/annual (as reported)	Establishment gate (microclimate vs coarse climate)	[6]
Mosquito	Aedes albopictus	Dengue/chikungunya (Europe)	Europe	Mixed temperate (Cfb/Dfb/Csa/Cfa)	Climate + vector establishment as predictors in a time-to-event framework	seasonal/annual (as reported)	Stage logic: establishment + climate → outbreak hazard	[7]
Tick	Ixodes ricinus	Borrelia spp.	UK (southern England)	Cfb (oceanic, dominant)	Urban–rural gradient context; environment/microclimate implicitly structured by land cover	seasonal/annual (as reported)	Avoid over-generalization (risk not monotonic with urbanization)	[8]
Tick	Ixodes ricinus	Thermal suitability	Europe (long-term)	Mixed temperate (Cfb/Dfb/Csa/Cfa)	Thermal limit shift (temperature-based suitability boundary)	seasonal/annual (as reported)	Range/phenology climate linkage	[9]
Tick	Ixodes scapularis	Borrelia burgdorferi s.s.	North America (residential–woodland gradient)	Temperate (mixed)	Fine-scale habitat gradient as proxy for microclimate exposure context	seasonal/annual (as reported)	Spillover interface (peri-domestic risk gradients)	[10]
Tick	Ixodes scapularis	Borrelia burgdorferi s.s.	North America	Temperate (mixed)	Host management effects; climate is not the sole driver	seasonal/annual (as reported)	Host specificity: deer affect ticks, not necessarily infection prevalence	[11]

Tick	Ixodes ricinus	Population dynamics	Europe (longitudinal field surveys)	Mixed temperate (Cfb/Dfb/Csa/Cfa)	Mechanistic modeling using climate covariates + field time series	multi-year	Driver precision (explicit climate terms in models)	[12]
Sandfly	Phlebotomus spp.	Distribution (France)	France (temperate margin + Mediterranean)	Cfb (oceanic) / Csa (mediterranean, south) / Dfb (NE)	Distribution/abundance synthesis; climate context discussed	seasonal/annual (as reported)	Specific geography (where sandflies occur in temperate France)	[13]
Sandfly	Phlebotomus mascittii	Presence updates	Southwest Germany	Cfb (oceanic) / Dfb (continental, parts)	Northern-edge occurrence; climate context (temperate margin)	seasonal/annual (as reported)	Establishment hypothesis (repeated detections at new locations)	[14]
Sandfly	Phlebotomus spp.	Toscana virus / Leishmania	Northern Italy (lowlands)	Cfa (humid subtropical, Po Valley) / Cfb (uplands)	Seasonal dryness/hydroclimate framing for abundance	seasonal/annual (as reported)	Amplification (hydroclimate as a seasonal amplifier)	[15]
Sandfly	Phlebotomus perniciosus	Toscana virus	Lab–mechanistic		Temperature-dependent intra-vector dynamics (EIP context)	seasonal/annual (as reported)	Pathogen-vector biology (omit vague statements)	[16]
Sandfly	(multiple)	Leishmania infantum autochthony (Europe)	Europe	Mixed temperate (Cfb/Dfb/Csa/Cfa)	Estimated distribution across 2005–2020; climate suitability context	seasonal/annual (as reported)	Where autochthony is supported	[17]
Biting midge	Culicoides spp.	Schmallenberg virus	Denmark (cattle farms)	Cfb (oceanic, dominant)	Microclimatic farm temperatures used to derive transmission potential	seasonal/annual (as reported)	Microclimate operationalization (farm-level)	[18]
Biting midge	Culicoides spp.	Flight activity constraints	UK	Cfb (oceanic, dominant)	Thermal limits for flight activity (lab-defined; field-collected)	experimental (temperature regimes)	Biology specificity (flight thresholds vary by complex)	[19]
Biting midge	Culicoides spp.	Bluetongue virus	(modeling; livestock context)		Temperature-dependent suitability of transmission	seasonal/annual (as reported)	Amplification window (temperature → suitability)	[20]
Biting midge	Culicoides spp.	BTV-3 / SBV	Germany	Cfb (oceanic) / Dfb (continental, parts)	Vector pool detection during epidemic context; seasonality implied	seasonal/annual (as reported)	Transmission-relevant surveillance (beyond "vector present")	[21]
Biting midge	Culicoides spp.	Winter activity	Germany (stables vs outdoors)	Cfb (oceanic) / Dfb (continental, parts)	Indoor–outdoor thermal buffering framing	winter; seasonal/annual (as reported)	Overwintering/persistence plausibility	[22]
Biting midge	Culicoides spp.	Bite prevention	Temperate farm setting	Temperate (varies)	Vector-proof accommodation effectiveness (exposure reduction)	seasonal/annual (as reported)	Control feasibility (farm-level intervention)	[23]
Mosquito	Culex spp.	West Nile virus	Europe (projection study)	Mixed temperate (Cfb/Dfb/Csa/Cfa)	Climate-change projections of WNV transmission suitability (temperature-driven, scenario-based)	future scenarios (multi-decade)	Provides map-based climate suitability/projection framing for temperate WNV risk	[24]
Mosquito	Culex spp.	West Nile virus	Modeling (temperature-driven transmission limits)		Temperature effects on boundary conditions of WNV transmission (mechanistic / model-based)	temperature-dependent; seasonal applicability	Strengthens the mechanistic link between temperature and WNV transmission potential	[25]
Mosquito	Culex spp.	West Nile virus	Europe	Mixed temperate (Cfb/Dfb/Csa/Cfa)	Climate-linked spatial expansion analysis (map-	multi-year (historical + recent)	Provides map-based evidence linking climate trends to WNV spatial expansion	[26]

					based); climatic suitability drivers			
Mosquito	Aedes albopictus	Overwintering ecology	Temperate urban microhabitats (tires)		Microhabitat thermal buffering (snow-covered tires) enables overwintering	winter	Adds concrete microclimate parameterization for overwintering survival	[27]
Tick	Ixodes ricinus	Microclimate constraints / ecological barriers	Europe (microclimate barrier study)	Mixed temperate (Cfb/Dfb)	Temperature and relative humidity patterns associated with ecological barriers to tick activity	seasonal / microclimate	Adds explicit temperature/RH covariates for tick persistence/establishment	[28]

References

1. Beck, H.E.; Zimmermann, N.E.; McVicar, T.R.; Vergopolan, N.; Berg, A.; Wood, E.F. Present and Future Köppen-Geiger Climate Classification Maps at 1-Km Resolution. *Sci. Data* **2018**, *5*, 180214, doi:10.1038/sdata.2018.214.
2. Farooq, Z.; Rocklöv, J.; Wallin, J.; Abiri, N.; Sewe, M.O.; Sjödin, H.; Semenza, J.C. Artificial Intelligence to Predict West Nile Virus Outbreaks with Eco-Climatic Drivers. *Lancet Reg. Health Eur.* **2022**, *17*, 100370, doi:10.1016/j.lanepe.2022.100370.
3. Cox, V.M.; Tiley, K.; Rosa, R.; Pugliese, A.; Angelini, P.; Carrieri, M.; Bhatt, S.; Tamba, M.; Marini, G.; Calzolari, M.; et al. Meteorological and Environmental Drivers of West Nile Virus Prevalence in Culex Pipiens Mosquitoes in Emilia-Romagna, Italy in 2013 to 2022. *PLOS Pathog.* **2025**, *21*, e1013753, doi:10.1371/journal.ppat.1013753.
4. Aguilera-Sepúlveda, P.; Napp, S.; Llorente, F.; Solano-Manrique, C.; Molina-López, R.; Obón, E.; Solé, A.; Jiménez-Clavero, M.Á.; Fernández-Pinero, J.; Busquets, N. West Nile Virus Lineage 2 Spreads Westwards in Europe and Overwinters in North-Eastern Spain (2017–2020). *Viruses* **2022**, *14*, doi:10.3390/v14030569.
5. Romiti, F.; Casini, R.; Del Lesto, I.; Magliano, A.; Ermenegildi, A.; Droghei, S.; Tofani, S.; Scicluna, M.T.; Pichler, V.; Augello, A.; et al. Characterization of Overwintering Sites (Hibernacula) of the West Nile Vector Culex Pipiens in Central Italy. *Parasit. Vectors* **2025**, *18*, 74, doi:10.1186/s13071-025-06710-5.
6. Ravasi, D.; Mangili, F.; Huber, D.; Cannata, M.; Strigaro, D.; Flacio, E. The Effects of Microclimatic Winter Conditions in Urban Areas on the Risk of Establishment for Aedes Albopictus. *Sci. Rep.* **2022**, *12*, 15967, doi:10.1038/s41598-022-20436-9.
7. Farooq, Z.; Segelmark, L.; Rocklöv, J.; Lillepold, K.; Sewe, M.O.; Briet, O.J.T.; Semenza, J.C. Impact of Climate and Aedes Albopictus Establishment on Dengue and Chikungunya Outbreaks in Europe: A Time-to-Event Analysis. *Lancet Planet. Health* **2025**, *9*, e374–e383, doi:10.1016/S2542-5196(25)00059-2.
8. Hansford, K.M.; McGinley, L.; Wheeler, B.W.; Tschirren, B.; Medlock, J.M. Ixodes Ricinus Density, Borrelia Prevalence and the Density of Infected Nymphs along an Urban–Rural Gradient in Southern England. *Zoonoses Public Health* **2023**, *70*, 304–314, doi:10.1111/zph.13024.
9. Da Re, D.; Gilson, G.F.; Dalaiden, Q.; Goosse, H.; Bødker, R.; Kjær, L.J.; Ogden, N.H.; Rosà, R.; Vanwambeke, S.O. Northward Expansion of the Thermal Limit for the Tick Ixodes Ricinus over the Past 40 Years. *Parasit. Vectors* **2025**, *18*, 449, doi:10.1186/s13071-025-07084-4.
10. Logan, J.J.; Knudby, A.; Leighton, P.A.; Talbot, B.; McKay, R.; Ramsay, T.; Blanford, J.I.; Ogden, N.H.; Kulkarni, M.A. Ixodes Scapularis Density and Borrelia Burgdorferi Prevalence along a Residential-Woodland Gradient in a Region of Emerging Lyme Disease Risk. *Sci. Rep.* **2024**, *14*, 13107, doi:10.1038/s41598-024-64085-6.
11. Martin, A.M.; Buttke, D.; Raphael, J.; Taylor, K.; Maes, S.; Parise, C.M.; Ginsberg, H.S.; Cross, P.C. Deer Management Generally Reduces Densities of Nymphal *Ixodes Scapularis*, but Not Prevalence of Infection with *Borrelia Burgdorferi* Sensu Stricto. *Ticks Tick-Borne Dis.* **2023**, *14*, 102202, doi:10.1016/j.ttbdis.2023.102202.
12. Kim, Y.; Jaulhac, B.; Vesga, J.F.; Zilliox, L.; Boulanger, N.; Edmunds, W.J.; Métras, R. Environmental Drivers of *Ixodes Ricinus* Tick Population Dynamics: Mechanistic Modelling Using Longitudinal Field Surveys and Climate Data. *Epidemics* **2025**, *53*, 100854, doi:10.1016/j.epidem.2025.100854.

13. Prudhomme, J.; Depaquit, J.; Robert-Gangneux, F. Phlebotomine Sand Fly Distribution and Abundance in France: A Systematic Review. *Parasite* **2024**, *31*, 45, doi:10.1051/parasite/2024045.
14. Oerther, S.; Jöst, H.; Heitmann, A.; Lühken, R.; Krüger, A.; Steinhausen, I.; Brinker, C.; Lorentz, S.; Marx, M.; Schmidt-Chanasit, J.; et al. Phlebotomine Sand Flies in Southwest Germany: An Update with Records in New Locations. *Parasit. Vectors* **2020**, *13*, 173, doi:10.1186/s13071-020-04058-6.
15. Calzolari, M.; Romeo, G.; Munari, M.; Bonilauri, P.; Taddei, R.; Sampieri, M.; Bariselli, S.; Rugna, G.; Dottori, M. Sand Flies and Pathogens in the Lowlands of Emilia-Romagna (Northern Italy). *Viruses* **2022**, *14*, doi:10.3390/v14102209.
16. Laroche, L.; Bañuls, A.-L.; Charrel, R.; Fontaine, A.; Ayhan, N.; Prudhomme, J. Sand Flies and Toscana Virus: Intra-Vector Infection Dynamics and Impact on Phlebotomus Perniciosus Life-History Traits. *PLoS Negl. Trop. Dis.* **2024**, *18*, e0012509, doi:10.1371/journal.pntd.0012509.
17. Maia, C.; Conceição, C.; Pereira, A.; Rocha, R.; Ortuño, M.; Muñoz, C.; Jumakanova, Z.; Pérez-Cutillas, P.; Özbel, Y.; Töz, S.; et al. The Estimated Distribution of Autochthonous Leishmaniasis by Leishmania Infantum in Europe in 2005–2020. *PLoS Negl. Trop. Dis.* **2023**, *17*, e0011497, doi:10.1371/journal.pntd.0011497.
18. Haider, N.; Cuellar, A.C.; Kjær, L.J.; Sørensen, J.H.; Bødker, R. Microclimatic Temperatures at Danish Cattle Farms, 2000–2016: Quantifying the Temporal and Spatial Variation in the Transmission Potential of Schmallenberg Virus. *Parasit. Vectors* **2018**, *11*, 128, doi:10.1186/s13071-018-2709-8.
19. Tugwell, L.A.; England, M.E.; Gubbins, S.; Sanders, C.J.; Stokes, J.E.; Stoner, J.; Graham, S.P.; Blackwell, A.; Darpel, K.E.; Carpenter, S. Thermal Limits for Flight Activity of Field-Collected Culicoides in the United Kingdom Defined under Laboratory Conditions. *Parasit. Vectors* **2021**, *14*, 55, doi:10.1186/s13071-020-04552-x.
20. El Moustaid, F.; Thornton, Z.; Slamani, H.; Ryan, S.J.; Johnson, L.R. Predicting Temperature-Dependent Transmission Suitability of Bluetongue Virus in Livestock. *Parasit. Vectors* **2021**, *14*, 382, doi:10.1186/s13071-021-04826-y.
21. Voigt, A.; Kampen, H.; Heuser, E.; Zeiske, S.; Hoffmann, B.; Höper, D.; Holsteg, M.; Sick, F.; Ziegler, S.; Wernike, K.; et al. Bluetongue Virus Serotype 3 and Schmallenberg Virus in Culicoides Biting Midges, Western Germany, 2023 - Volume 30, Number 7—July 2024 - Emerging Infectious Diseases Journal - CDC. **2024**, doi:10.3201/eid3007.240275.
22. Groschupp, S.; Kampen, H.; Werner, D. Winter Activity of Culicoides (Diptera: Ceratopogonidae) inside and Outside Stables in Germany. *Med. Vet. Entomol.* **2024**, *38*, 552–565, doi:10.1111/mve.12756.
23. King, S.; Nicholls, M.; Scales, J.; Gubbins, S.; Pearce-Kelly, P.; Saverimuttu, S.; Forsyth, S.; England, M. The Efficacy of Vector-Proof Accommodation for the Protection of Livestock against Culicoides Biting Midges. *Parasit. Vectors* **2025**, *18*, 108, doi:10.1186/s13071-025-06736-9.
24. McPake, B.; Gilbert, K.; Vong, S.; Ros, B.; Has, P.; Khuong, A.T.; Phuc, P.-D.; Hoang, Q.C.; Nguyen, D.H.; Siengsounthone, L.; et al. Role of Regulatory Capacity in the Animal and Human Health Systems in Driving Response to Zoonotic Disease Outbreaks in the the Mekong Region. *One Health Amst. Neth.* **2022**, *14*, 100369, doi:10.1016/j.onehlt.2022.100369.
25. de Freitas Costa, E.; Streng, K.; Avelino de Souza Santos, M.; Counotte, M.J. The Effect of Temperature on the Boundary Conditions of West Nile Virus Circulation in Europe. *PLoS Negl. Trop. Dis.* **2024**, *18*, e0012162, doi:10.1371/journal.pntd.0012162.
26. Erazo, D.; Grant, L.; Ghisbain, G.; Marini, G.; Colón-González, F.J.; Wint, W.; Rizzoli, A.; Van Bortel, W.; Vogels, C.B.F.; Grubaugh, N.D.; et al. Contribution of Climate Change to the Spatial Expansion of West Nile Virus in Europe. *Nat. Commun.* **2024**, *15*, 1196, doi:10.1038/s41467-024-45290-3.
27. Susong, K.M.; Tucker, B.J.; Bron, G.M.; Irwin, P.; Kirsch, J.M.; Vimont, D.; Stone, C.; Paskewitz, S.M.; Bartholomay, L.C. Snow-Covered Tires Generate Microhabitats That Enhance Overwintering Survival of Aedes Albopictus (Diptera: Culicidae) in the Midwest, USA. *Environ. Entomol.* **2022**, *51*, 586–594, doi:10.1093/ee/nvac023.
28. Grigoryeva, L.A. Influence of Air Humidity on the Survival Rate, Lifetime, and Development of Ixodes Ricinus (L., 1758) and Ixodes Persulcatus Schulze, 1930 (Acari: Ixodidae). *Syst. Appl. Acarol.* **2022**, *27*, 2241–2248, doi:10.11158/saa.27.11.9.
